# Cholera intoxication of human enteroids reveals interplay between decoy and functional glycoconjugate ligands

**DOI:** 10.1093/glycob/cwad069

**Published:** 2023-08-25

**Authors:** Akshi Singla, Andrew Boucher, Kerri-Lee Wallom, Michael Lebens, Jennifer J Kohler, Frances M Platt, Ulf Yrlid

**Affiliations:** Department of Microbiology and Immunology, Institute of Biomedicine, University of Gothenburg, Medicinaregatan 1G, 41390 Gothenburg, Sweden; Department of Medical Chemistry and Cell Biology, Institute of Biomedicine, University of Gothenburg, Medicinaregatan 1G, 41390 Gothenburg, Sweden; Department of Microbiology and Immunology, Institute of Biomedicine, University of Gothenburg, Medicinaregatan 1G, 41390 Gothenburg, Sweden; Department of Pharmacology, University of Oxford, Mansfield Road, Oxford OX1 3QT, United Kingdom; Department of Microbiology and Immunology, Institute of Biomedicine, University of Gothenburg, Medicinaregatan 1G, 41390 Gothenburg, Sweden; Department of Biochemistry, University of Texas Southwestern Medical Center, 5323 Harry Hines Blvd., Dallas, TX 75390-9185, United States; Department of Pharmacology, University of Oxford, Mansfield Road, Oxford OX1 3QT, United Kingdom; Department of Microbiology and Immunology, Institute of Biomedicine, University of Gothenburg, Medicinaregatan 1G, 41390 Gothenburg, Sweden

**Keywords:** cholera toxin, decoy like ligands, enteroid monolayers, fucosylation, O-glycosylation

## Abstract

Prior research on cholera toxin (CT) binding and intoxication has relied on human colonic cancer derived epithelial cells. While these transformed cell lines have been beneficial, they neither derive from small intestine where intoxication occurs, nor represent the diversity of small intestinal epithelial cells (SI-ECs) and variation in glycoconjugate expression among individuals. Here, we used human enteroids, derived from jejunal biopsies of multipledonors to study CT binding and intoxication of human non-transformed SI-ECs. We modulated surface expression of glycosphingolipids, glycoproteins and specific glycans to distinguish the role of each glycan/glycoconjugate. Cholera-toxin-subunit-B (CTB) mutants were generated to decipher the preference of each glycoconjugate to different binding sites and the correlation between CT binding and intoxication. Human enteroids contain trace amounts of GM1, but other glycosphingolipids may be contributing to CT intoxication. We discovered that inhibition of either fucosylation or O-glycosylation sensitize enteroids to CT-intoxication. This can either be a consequence of the removal of fucosylated “decoy-like-ligands” binding to CTB’s non-canonical site and/or increase in the availability of Gal/GalNAc-terminating glycoconjugates binding to the canonical site. Furthermore, simultaneous inhibition of fucosylation and O-glycosylation increased the availability of additional Gal/GalNAc-terminating glycoconjugates but counteracted the sensitization in CT intoxication caused by inhibiting O-glycosylation because of reduction in fucose. This implies a dual role of fucose as a functional glycan and a decoy, the interplay of which influences CT binding and intoxication. Finally, while the results were similar for enteroids from different donors, they were not identical, pointing to a role for human genetic variation in determining sensitivity to CT.

## Introduction

An estimated 2.9 million cholera cases occur annually in 69 endemic countries, with most of the burden in the Global South (Ali et al. 2015). Cholera toxin (CT), secreted by the bacteria *Vibrio cholerae,* is an AB_5_ multimeric protein comprising of an enzymatically active A subunit separated from the plane of a ring formed by a pentameric binding B subunit. In the small intestinal lumen, CT binds to epithelial cells of the small intestine via the B subunit (CTB), followed by retrograde transport to the endoplasmic reticulum and retrotranslocation of the subunit A (CTA) to the cytoplasm. Through its ADP-ribosylation activity, CTA causes chloride ion efflux and osmotic imbalance. This results in diarrhea with extensive loss of water and electrolytes that could threaten the life of the infected individual if untreated (Sánchez and Holmgren 2008). Since binding of CT to the host cell epithelium is the primary step, identifying these binding mechanisms can increase the understanding of the cholera pathogenesis and possibly assist in the development of antidotes and improved vaccines. However, the underlying principles that govern the binding of CT to primary human small intestinal cells and its influence on cellular intoxication remain enigmatic.

The binding affinity between CTB and GM1 glycosphingolipid is extremely high and consequently GM1 has been considered the initial binding target of CT and the first step of human intestinal intoxication for more than forty years. However, recent studies have questioned the significance of GM1 in CT intoxication because of a) scarce GM1 density on human small intestinal epithelial cells (<0.003 mol% of the total glycosphingolipids) (Breimer et al. 2012); b) poor correlation between CTB binding and GM1 density (Yanagisawa et al. 2006; Kirkeby 2014). In addition, we have previously shown that mice deficient in synthesis of GM1 and GM1 related glycosphingolipids remain sensitive to CT-induced intoxication (Cervin et al. 2018). The severity of cholera infection has also been associated with the differential expression of fucosylated histo-blood group antigens in the human gastrointestinal tract (Swerdlow et al. 1994; Harris et al. 2008; Heggelund et al. 2012; Heggelund et al. 2016). Furthermore, we have recently shown that CTB binds to fucosylated structures on colonic epithelial cell lines (Wands et al. 2015, 2018; Sethi et al. 2019) and a mixture of fucose and galactose on a polymer backbone could also inhibit CTB binding and reduce CT intoxication more efficiently than only galactose (Cervin et al. 2020). Thus, there is now ample evidence showing that CT binding can be GM1-independent and that alternative glycoconjugates can influence this process. Importantly, crystallographic studies have revealed the presence of a secondary non-canonical binding site other than the canonical GM1 binding site on CTB, which can have significance for CT binding (Heggelund et al. 2016; Heim et al. 2019).

Human colon cancer-derived epithelial cell lines such as T84, Caco-2, Colo205 and HT29 cells have primarily been used to explore CT binding process. However, cell lines derived from a single donor do not mimic the diversity of epithelial cells in the intestine (Huang et al. 2021). The surface glycome has also been shown to be altered upon transformation and genetic heterogeneity may arise with increasing passage number or non-uniform subculturing practices (Hughes et al. 2007; Kaur and Dufour 2012). Moreover, the glycan composition varies from the small intestine to the colon (Robbe et al. 2003; Holmén Larsson et al. 2013; Ringot-Destrez et al. 2018) and *Vibrio cholerae* has been shown to colonize the small intestine (Millet et al. 2014; Almagro-Moreno et al. 2015). The non-transformed human intestinal explants overcome these limitations, but can only be used for short term and need to be continuously sourced (Ranganathan et al. 2020).

Human small intestinal organoids (enteroids) offer an excellent platform for investigation of bacterial binding and intoxication processes as they recapitulate normal intestinal physiology, retain donor genetics as well as intestinal epithelial diversity, segmental specification, cell polarization, barrier function and mucus secretion (Sato and Clevers 2013; Zachos et al. 2016; Ranganathan et al. 2020). Human enteroids have also been used to characterize electrolyte or ion transport in diarrhea-like conditions and thus they have functional relevance to intestinal ion transport pathophysiology (Foulke-Abel et al. 2016; Cervin et al. 2020). Recently, enteroids were used to demonstrate blood group dependent cellular responses to CT, which aligns well with prior epidemiological studies (Kuhlmann et al. 2016). However, non-transformed human small intestinal epithelium has been very rarely used to elucidate the influence of different glycoconjugates in CT binding and even more rarely in intoxication.

In this study, we have therefore used human enteroids, derived from jejunal biopsies from multiple donors, to methodically investigate the role of glycosphingolipids and glycoproteins as well as specific groups of glycans in CT binding to and intoxication of human small intestinal epithelial cells. To facilitate this analysis, we created CTB mutants with different functional activity in each binding site to decipher the preference of each glycoconjugate in CTB binding and study the correlation between binding and intoxication. We determined that the amount of GM1 in human small intestinal enteroids is very scant. We suspect an important role of “decoy-like-ligands” in CT binding and intoxication i.e. ligands on host cells that can bind to CTB, but do not promote CT intoxication. We revealed that inhibition of fucosylation as well as O-linked glycosylation sensitized the enteroids to intoxication, which could be due to removal of “decoy-like-ligands” that bind via the non-canonical site of CTB and/or increase in surface availability of Gal/GalNAc terminated glycoconjugates that bind CTB via the canonical site and functionally contribute to CT-mediated intoxication. Glycosphingolipids other than GM1 may be contributing to CT intoxication, however their function gets revealed when O-glycosylation is inhibited which could be because of easier accessibility in the absence of “decoy-like-ligands.” We identified that fucose is an important glycan as it may have a functional role in CT intoxication and/or contributing to CT binding to “decoy-like-ligands.” This could also explain the severity of cholera infections in individuals with O blood group that displays terminal fucosylated H-antigen (Swerdlow et al. 1994; Mandal et al. 2012; Kuhlmann et al. 2016). Hence, this study has started to unravel the functional and sometimes opposing roles of different glycoconjugates on non-transformed human small intestinal cells that CTB binds to.

## Results

### Role of glycosphingolipids in CT intoxication of enteroids

Prior literature has demonstrated that binding of cholera toxin to GM1 has the highest binding affinity whereas binding to other glycosphingolipids such as fucosyl-GM1, GD1b, GD1a, GT1b, asialo-GM1, GM2, GM3, occurs at lower binding affinities (Kuziemko et al. 1996; Lauer et al. 2002; Turnbull et al. 2004; Han et al. 2016; Krishnan et al. 2017; Lee et al. 2018). Thus, to investigate the role of glycosphingolipids in CT-induced ion efflux (intoxication) of the human small intestine, confluent monolayers of human enteroids derived from jejunal biopsies of four different donors were established. On the third day of differentiation, a selective inhibitor of glycosphingolipid biosynthesis, NB-DGJ (Platt et al. 1994), was introduced in the differentiation media for 72 h prior to apical challenge with CT.

**Fig. 1:**
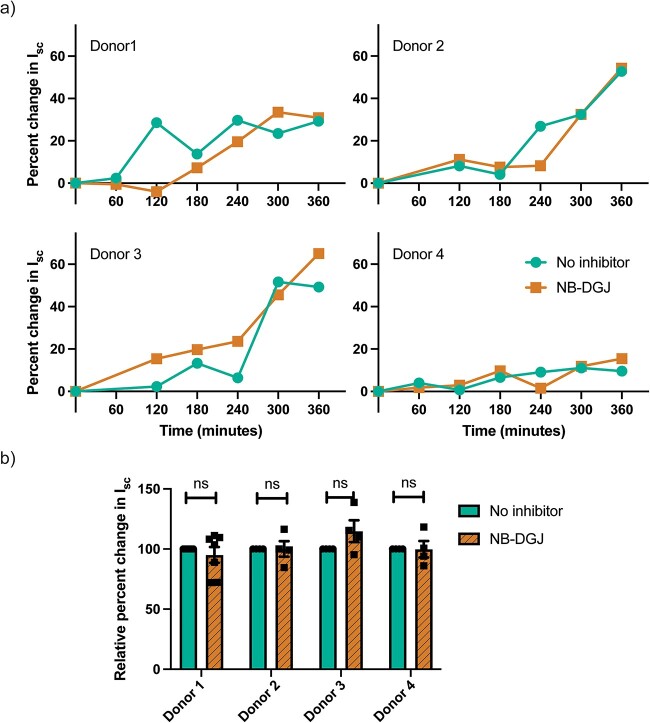
Role of glycosphingolipids in CT intoxication of human enteroids. The CT induced ion efflux (intoxication) is determined by estimating the change in short circuit current from transmembrane voltage and resistance measurements in enteroids cultured with or without 50 μM NB-DGJ, that inhibits glycosphingolipid (including GM1) biosynthesis, for 72 h prior to CT challenge. a) Rate of CT intoxication in enteroid monolayers from jejunal biopsies from four different donors. The data shown is a representative of *n* ≥ 4 independent experiments of each enteroid donor. b) Pooled data of normalized change in short circuit current (Isc) i.e. Isc normalized to when no NB-DGJ is present, 5 h after apical treatment with CT. Data is reported as mean ± S.E. (*n* ≥ 4 biological replicates). n.s (*p* > 0.05).

The sensitivity to CT differed between enteroids derived from the different donors but importantly for none of them did the presence of NB-DGJ (inhibition of glycosphingolipid biosynthesis) affect the CT intoxication to a significant degree (Fig. 1). Furthermore, we determined the amount of CTB binding to human enteroids in the presence of 50 µM NB-DGJ and 1 µM P4 (Abe et al. 1992), another inhibitor of glucosylceramide synthetase, using flow cytometry. P4 is a more potent inhibitor than NB-DGJ, however, P4 was toxic to enteroids. Neither of the glycosphingolipid biosynthesis inhibitors reduced the amount of CTB binding to human enteroids (Fig. S1a). However, when the GM1-rich Jurkat cells were grown with either NB-DGJ or P4, the amount of GM1 as well as CTB binding was significantly reduced (Figs S1b and S2).

To evaluate the efficacy of NB-DGJ, we assessed the expression of glycosphingolipids in human enteroids before and after NB-DGJ treatment. While NB-DGJ treatment reduced the levels of glycosphingolipids, the expression of glycosphingolipids was not eliminated entirely (Fig. S3a). Thus, the functional role of glycosphingolipids in CT intoxication cannot be disregarded. However, GM1 was not detected even before NB-DGJ treatment and the amount of other known low-affinity ligands of CTB such as GM2 and GM3 was less than 3.4% which was further reduced after the NB-DGJ treatment (Fig. S3b and c). This provides further evidence that despite GM1 having the highest binding affinity for CTB, this may not significantly contribute to CT intoxication because of low surface expression on non-transformed human small intestinal epithelial cells (Wands et al. 2015; Cervin et al. 2018). Additionally, significant presence of Lewis-b in enteroids from only one donor implies that one donor is a secretor while the other is not (Fig. S3b and c) (Stowell and Stowell 2019).

### Role of fucosylation in CT intoxication of human enteroids

**Fig. 2:**
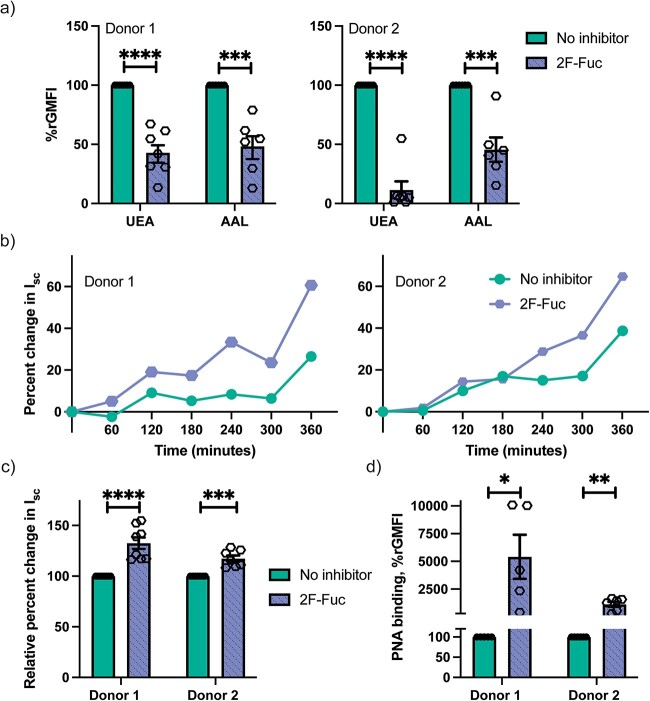
Role of fucosylation in CT intoxication of human enteroids. a) Relative fucosylation-dependent binding of lectin UEA-1 and AAL is reduced in the presence of 200 μM 2F-Fuc, fucosylation inhibitor. To statistically analyze the multiple biological replicates, the relative geometric mean fluorescence intensity (rGMFI) is determined by normalizing the GMFI for each condition to the GMFI when no inhibitor is present in the same experiment. Data is reported as mean ± S.E. (*n* ≥ 6 biological replicates). b) The CT induced ion efflux (intoxication) is determined by estimating the change in short circuit current from transmembrane voltage and resistance measurements in enteroids treated with or without 200 μM 2F-Fuc. The data shown is a representative of *n* ≥ 7 independent experiments. c) Normalized change in short circuit current (Isc) after 5 h of apical treatment with CT. Inhibition of fucosylation increases the CT intoxication levels. Data is reported as mean ± S.E. (*n* ≥ 7 biological replicates). d) Gal/GalNAc dependent PNA binding to human enteroids is significantly increased in the presence of 2F-Fuc. Data is reported as mean ± S.E. (*n* ≥ 5 biological replicates). p value: ≥0.05 (ns), <0.05 (^*^), <0.01(^*^^*^), <0.001(^*^^*^^*^) and <0.0001(^*^^*^^*^^*^).

We have recently demonstrated that fucosylated glycoconjugates are primary ligands for CTB binding and CT intoxication in T84 cells, a human colonic epithelial cell line (Wands et al. 2015; Wands et al. 2018; Sethi et al. 2019). Therefore, we next investigated if fucosylated glycans have a similar role in human enteroids. The human enteroid differentiation media was supplemented with 2F-Fuc (Rillahan et al. 2012), a metabolic inhibitor of fucosylation, for 3 days before treatment with CT or CTB. 2F-Fuc reduced the amount of fucosylated glycans expressed on the human enteroids as the amount of two fucose binding lectins, UEA-1 (binds to α-1,2 linked fucose) and AAL (binds to α-1,3 and α-1,6 linked fucose) were significantly reduced (Fig. 2a). Contrary to results previously observed for T84 cells (Wands et al. 2015; Sethi et al. 2019), inhibition of fucosylation resulted in sensitization of the enteroids to CT-mediated intoxication (Fig. 2b and c). This suggests that human small intestinal enteroids, in contrast to T84 cells, express fucosylated glycoconjugates that act as “decoy-like-ligands” and consequently upon removal, increase the sensitivity to CT intoxication. Another possible contribution to this unexpected finding was unraveled when the surface expression of galactosylated ligands were assessed and showed that 2F-Fuc treatment results in a significant increase in binding of the lectin PNA that preferentially binds to galactose (Gal) and galactosyl-β-1,3-N-acetylgalactosamine (Galβ1-3GalNAc) structures (Fig. 2d). This suggests that 2F-Fuc treatment of human enteroids could also increase the surface expression of Gal/GalNAc terminated glycoconjugates that are primary ligands for CT binding as well as intoxication.

### Binding of CTB mutants with human enteroids

CTB binds to GM1 via the canonical site at extremely high binding affinity (Kd < 60 nM) whereas the CTB binding affinity to Lewis-x (Galβ(1–4)[Fucα(1–3)]GlcNAc) via the non-canonical site is at least five orders of magnitude lower (Kd 8 mM) (Worstell et al. 2016; Heim et al. 2019). To elucidate the role of the two groups of potential surface ligands on human enteroids we synthesized site specific CTB mutants (Fig. S4), WT (both binding sites are intact), W88K (impaired canonical binding site), H18L (impaired non-canonical binding site) and H18LW88K (both binding sites are impaired) (Jobling and Holmes 1991).

**Fig. 3:**
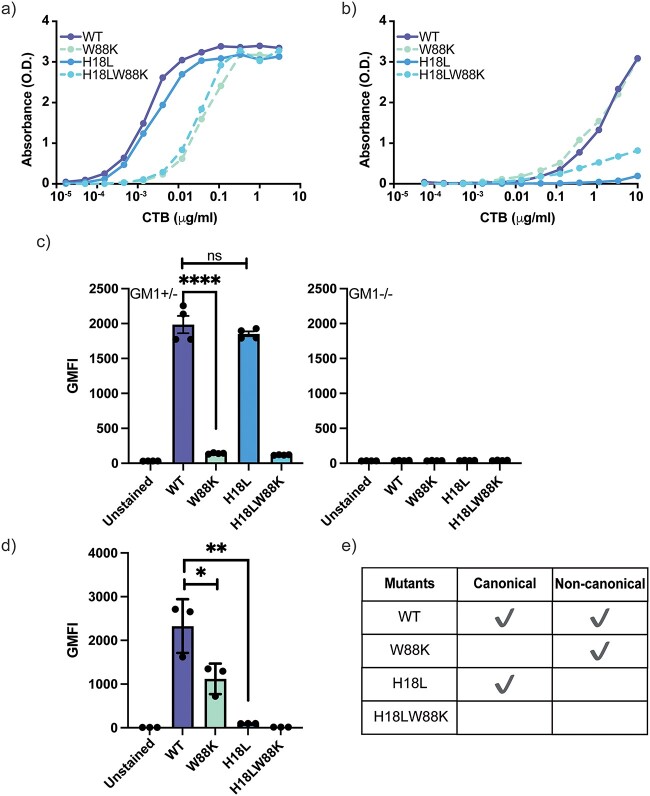
Binding of site-specific CTB mutants to a) plate-immobilized GM1-HSA (0.05 μg mL^−1^) as assessed by ELISA, b) plate-immobilized tri-Lewis-x-APE-HSA (1 μg mL^−1^) as assessed by ELISA, c) murine splenic T cells from wild-type mice (GM1+/−) and B4galnt1 knockout mice (GM1−/−) as assessed by flow cytometry, and d) human granulocytes as assessed by flow cytometry. a, b) One representative of three independent experiments is shown. c, d) Data is reported as mean ± S.E. (*n* ≥ 3 biological replicates) of geometric mean fluorescence intensity (GMFI). p value: ≥0.05 (ns), <0.05 (^*^), <0.01(^*^^*^), <0.001(^*^^*^^*^), and <0.0001(^*^^*^^*^^*^).

First, we verified the efficacy of the mutations by evaluating WT, W88K, H18L and H18LW88K binding with plate immobilized GM1 and Lewis-x in ELISA. H18L binding to GM1 was comparable to that of WT whereas the W88K binding to GM1 was considerably reduced (Fig. 3a). In contrast, W88K and WT bound to Lewis-x with similar efficacy while H18L hardly bound to Lewis-x (Fig. 3b). As expected, H18LW88K showed significant reduction in binding to either ligand (Fig. 3a and b). We next assessed the binding of the CTB mutants to ligands on primary cells. It has previously been shown that CTB binding to murine splenocytes is almost entirely blocked by GM1oligosaccharides but not by Lewis-x oligosaccharides (Cervin et al. 2018), suggesting that the canonical binding site is being employed predominantly. Thus, we measured the binding of CTB mutants to T cells among isolated splenocytes from β1,4-N-acetyl-galactosaminyl transferase B4galnt1 knockout mice (GM1^−/−^) and heterozygous (GM1^+/−^) C57BL/6 mice (Fig. 3c). B4 is an essential enzyme involved in the biosynthesis of glycosphingolipids (GM1, GD1, GT1, and GA1). Our observation and previous research have shown very similar glycosphingolipid expression in homozygous (GM1^+/+^) and heterozygous (GM1^+/−^) mice (Takamiya et al. 1996). W88K almost entirely lost binding to GM1^+/−^ murine splenocytes, while H18L binding was not affected compared to WT CTB. At the same time, neither WT CTB nor any of the mutants bound to GM1^−/−^ splenocytes deficient in GM1 and GM1 like glycosphingolipids, confirming that CTB binds via the canonical site to these glycosphingolipids on murine T cells. On the other hand, CTB binding to human granulocytes has previously been shown to be hampered by blocking with Lewis-x-os but also affected by the addition of GM1-os (Cervin et al. 2018). We observed that the H18L binding to the human granulocytes was dramatically reduced compared to WT and W88K binding was reduced to almost half of WT (Fig. 3d). Thus, the binding capacity of the mutants were site-specific to ligands immobilized on a plate as well as targets expressed on the surface of primary cells.

**Fig. 4:**
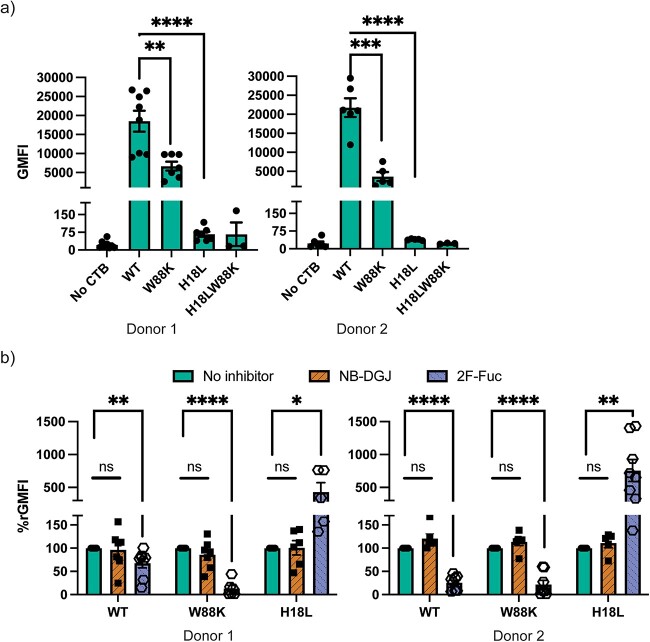
a) Binding of site-specific CTB mutants to human enteroids. Data is reported as mean ± S.E. (*n* ≥ 4 biological replicates) of GMFIs measured by flow cytometry. b) Binding of CTB mutants to human enteroids with and without inhibitors, 50 μM NB-DGJ (inhibition of glycosphingolipid synthesis) and 200 μM 2F-Fuc (inhibition of fucosylation). Data is reported as mean ± S.E. (*n* ≥ 5 biological replicates) of relative GMFI percentage (%rGMFI) where GMFI is normalized such that the measured GMFI with each mutant when no inhibitor is present is considered 100%. p value: ≥ 0.05 (ns), <0.05 (^*^), <0.01(^*^^*^), <0.001(^*^^*^^*^), and <0.0001(^*^^*^^*^^*^).

Thereafter, we explored the CTB mutants binding to human enteroids. H18L, with an impaired non-canonical site, almost entirely lost binding to the human enteroids from two donors (Fig. 4a). This implies that most of the ligands on human enteroids are bound to CTB via the non-canonical site. The binding of W88K to the enteroids is one fourth of the overall binding WT CTB, which suggests that the canonical site also, but to a lesser degree, binds to ligands on human enteroids.

Next, we assessed the binding of CTB mutants to human enteroids following inhibition of fucosylation or glycosphingolipid biosynthesis. Earlier, we observed that as fucosylation was inhibited, CT intoxication increases which could be because of reduction of fucosylated “decoy-like-ligands” and/or increased binding to Gal/GalNAc terminated glycoconjugates (Fig. 2b–d). However, when fucosylation was inhibited, the overall binding of WT CTB was reduced, suggesting that CTB binds to fucosylated ligands on human enteroids (Fig. 4b). In addition, these fucosylated ligands are bound through the non-canonical site as the residual binding by W88K (lacking canonical site) is entirely lost in the absence of fucose. On the other hand, an increased binding through the canonical site in H18L (deficient in non-canonical binding), was detected when fucosylation was inhibited (Fig. 4b). This correlates with the enhancement in the terminal Gal/GalNAc specific PNA binding (Fig. 2d). This implies that CT or CTB binding is not necessarily correlated with CT intoxication i.e. some glycoconjugates may bind to CTB but may not effectively mediate CT internalization. It also aligns well with the evidence from the literature that most of the fucosylated glycoconjugates bind to the non-canonical site while the galactosylated ligands bind to the canonical site (Ångström et al. 2000; Holmner et al. 2004; Holmner et al. 2007; Mandal et al. 2012; Vasile et al. 2014; Heggelund et al. 2016). It seems likely that galactosylated ligands are playing a more functional role in CT intoxication compared to fucosylated ligands. In contrast to 2F-Fuc, there were no significant differences in binding observed with any mutants following the treatment with NB-DGJ (Fig. 4b).

Hence these results show that human enteroids express ligands that CTB binds via the canonical as well as the non-canonical site. Since inhibition of fucosylation resulted in enhancement in CT intoxication, it is likely that the fucosylated ligands mediating binding to the non-canonical site act as “decoy-like-ligands” while the Gal/GalNAc terminated glycoconjugates binding to the canonical site are functional ligands.

### Role of glycoproteins in CT binding and intoxication to human enteroids

**Fig. 5:**
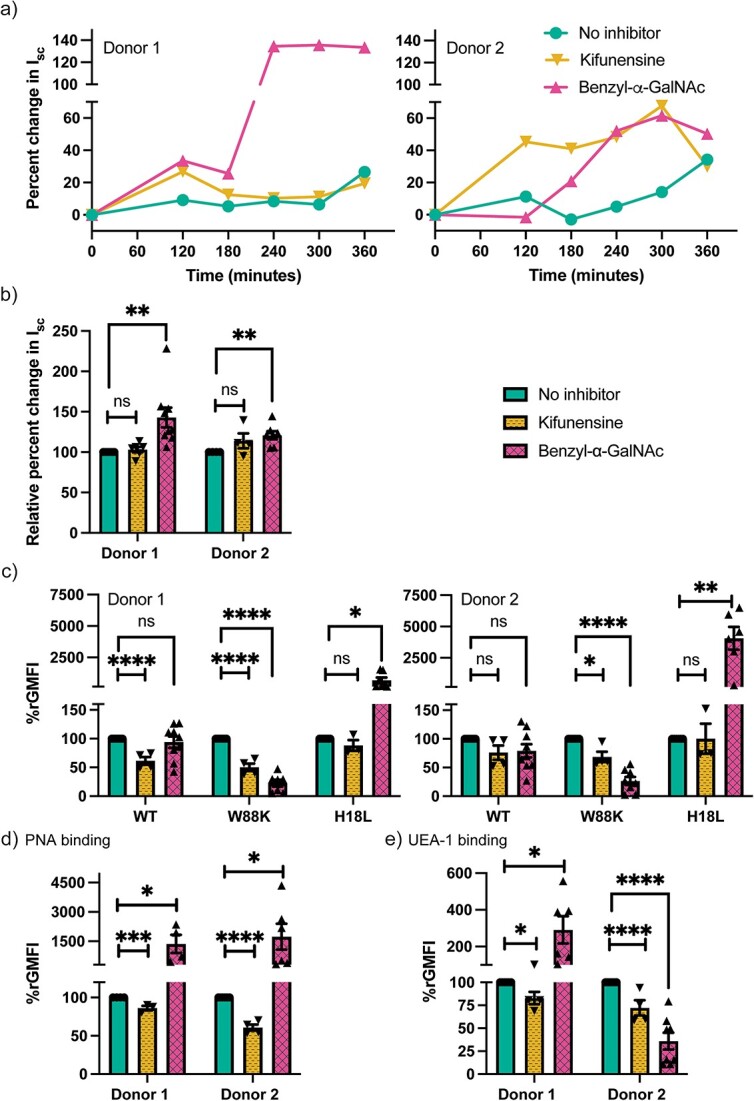
CT binding to and intoxication of human enteroids following inhibition of O-linked (2 mM benzyl-α-GalNAc) or N-linked (10 μM kifunensine) glycosylation. a) Rate of CT-mediated intoxication of enteroid monolayers. The data shown is a representative of *n* ≥ 4 independent experiments. b) Pooled data of normalized change in short circuit current (Isc) i.e. Isc normalized to when no inhibitor is present, 5 h after apical treatment with CT. Data is reported as mean ± S.E. (*n* ≥ 4 biological replicates). Binding of c) CTB mutants, d) PNA (galactose binding), e) UEA-1 (fucose binding) to human enteroids with and without benzyl-α-GalNAc and kifunensine measured via flow cytometry. Data is reported as mean ± S.E. (*n* ≥ 4 biological replicates) of relative GMFI percentage (%rGMFI) where GMFI is normalized such that the measured GMFI with each mutant when no inhibitor is present is considered as 100%. p value: ≥ 0.05 (ns), <0.05 (^*^), <0.01(^*^^*^), <0.001(^*^^*^^*^) and <0.0001(^*^^*^^*^^*^).

In addition to glycosphingolipids as surface ligands for CTB, glycoproteins have also been put forward as potential functional binding partners for CTB during the initial interactions with human colonic epithelial cells and rat small intestinal microvillus membranes intoxication (Morita et al. 1980; Wands et al. 2015). However, the nature of the glycosylated structures attached to potential protein ligands for CTB on human primary small intestinal epithelial cells has not been determined. Therefore, we next explored the role of O-linked and N-linked glycoproteins in CT-mediated intoxication of enteroids. For this, kifunensine was used to inhibit maturation of N-linked glycans and Benzyl-α-GalNAc was used as a decoy substrate to interfere with O-linked glycan biosynthesis. N-linked glycoproteins do not contribute significantly to CT intoxication even though some N-linked glycoproteins bind to the non-canonical site of CTB (Fig. 5a–c). However, inhibition of O-linked glycoproteins repeatedly resulted in a substantial upsurge in CT intoxication (Fig. 5a). This suggests that O-linked glycoproteins could act as “decoy-like-ligands” that limit the binding of CT to primary functional glycoconjugates and thereby functional ligands for CTB become accessible when O-glycosylation is inhibited. Alternatively/in addition to this, the cause of enhanced CT intoxication could be the increase in terminal Gal/GalNAc, as detected by PNA (Fig. 5d), caused by Benzyl-α-GalNAc that by its mode of action results in production of truncated glycans such as T or Tn antigens (Huet et al. 1995).

Furthermore, inhibition of O-glycosylation caused a reduction in the non-canonical site binding as demonstrated by the reduced binding of W88K (Fig. 5c). However, this reduction in binding to the non-canonical site was compensated by the substantial increment in the binding to the canonical site of H18L, such that the overall binding to WT CTB was statistically unaltered (Fig. 5c). This further substantiates the argument that O-linked glycoproteins might be binding to the non-canonical site of CTB wherein acting as decoy-like-ligands however, truncated ligands after the Benzyl-α-GalNAc treatment might be binding to the canonical site and contributing to CT intoxication. Additionally, inhibition of O-linked glycoproteins resulted in increased binding of fucose-specific lectins in enteroids from one donor whereas a decrease was observed in the other (Fig. 5e). These donor-specific alterations in expression of fucosylated structures (potential ligands for CTB) could be linked to human blood group antigens, as only one of the two donors appears to be a secretor (Fig. S3b and c).

Previous literature has reported that Benzyl-α-GalNAc treatment could also alter sialylation (Ulloa et al. 2000). We observed an increase in the α-2,6 linked sialylated ligands, as detected by SNA binding, but no difference in the α-2,3 linked sialylated ligands, as detected by MAL II binding (Fig. S5a). Even though the crystal structure shows a supporting role of sialic acid in CTB binding to GM1 (Merritt et al. 1994), CTB binds to sialic acid itself (Kd 210 mM) and other sialylated glycosphingolipids at much lower binding affinity (Fukuta et al. 1988; Turnbull et al. 2004; Krishnan et al. 2017). Our glycosphingolipid analysis shows no increase in GM1 or any other sialylated glycosphingolipids in response to Benzyl-α-GalNAc treatment (Fig. S8). To verify if changes in sialylation could cause non-specific CTB binding because of surface charge alterations, we assessed H18LW88K CTB-mutant (both binding sites are inactive) to human enteroids and observed no increase in binding upon Benzyl-α-GalNAc treatment (Fig. S5b). In addition, if the charge alterations had been the primary reason, the increase in CT intoxication upon Benzyl-α-GalNAc treatment should have correlated with increase in CTB WT binding to enteroids which was, however, not observed. Furthermore, we also modulated sialylation using *Vibrio cholerae* neuraminidase (VcN) which resulted in increase in both SNA and CTB WT binding to Jurkat cells (Fig. S6a). The increased CTB binding in Jurkat cells could be due to the removal of terminal sialic acid from polysialylated glycosphingolipids such as GD1a (Fig. S2), revealing GM1 (Alisson-Silva et al. 2018). However, VcN treatment to human enteroids caused a slight decrease in CTB WT binding, even though SNA binding increased, if anything (Fig. S6b and c). None of the other CTB mutants binding to human enteroids were altered upon VcN treatment. Therefore, even though there was an increase in sialylated ligands, it seems unlikely that sialylated ligands are playing a major role in CTB binding to human enteroids.

Thus, the use of Benzyl-α-GalNAc fulfilled a dual purpose by revealing a decoy like function of O-linked glycoproteins as well as potentially sensitizing the enteroids to synthesize a greater number of higher affinity functional glycoproteins which contribute to CT intoxication.

### Do glycosphingolipids play a more important role in the absence of decoys?

**Fig. 6:**
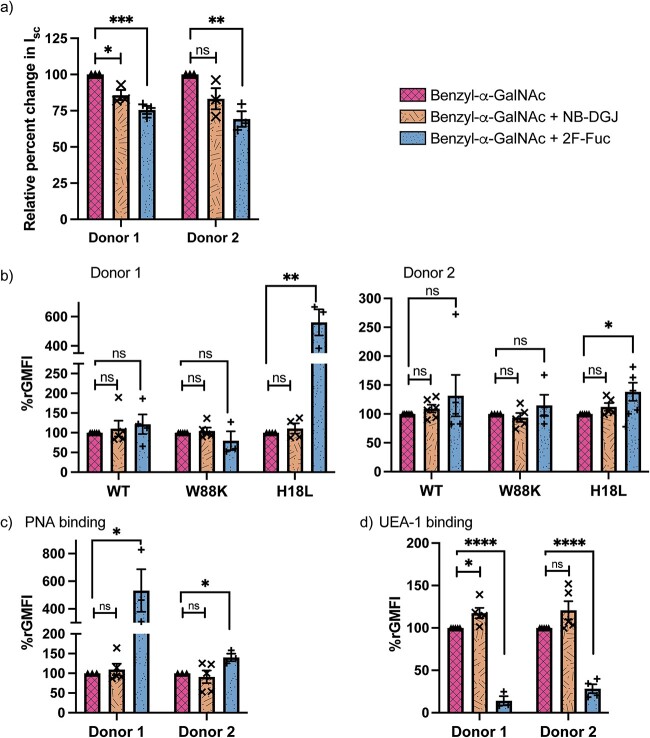
Role of glycosphingolipids and fucosylation in the absence of “decoy-like-ligands.” a) CT intoxication, Isc normalized to when only benzyl-α-GalNAc is present, 5 h after apical treatment with CT. Data is reported as mean ± S.E. (*n* = 3 biological replicates). b) Binding of CTB mutants to human enteroids measured via flow cytometry. Data is reported as mean ± S.E. (*n* ≥ 4 biological replicates) of relative GMFI percentage (%rGMFI) where GMFI is normalized such that the measured GMFI with each mutant when only Benzyl-α-GalNAc is present is considered as 100%. p value: ≥ 0.05 (ns), <0.05 (^*^), and <0.0001(^*^^*^^*^^*^).

We next wished to assess whether the observed sensitization of CT-induced intoxication of enteroids following O-glycosylation inhibition could be attributed to increased access to ligands expressed by glycosphingolipids. Therefore, human enteroids were cultured with Benzyl-α-GalNAc as well as Benzyl-α-GalNAc + NB-DGJ. As we previously observed (Fig. 5), O-glycosylation-inhibited enteroids i.e. enteroids treated with Benzyl-α-GalNAc were sensitized to CT intoxication, while a similar enhancement could not be detected when measuring WT CTB binding to human enteroids. In addition, the use of site-specific mutants unraveled the alteration in binding at each site and thereby the modified expression of galactosylated and fucosylated surface ligands. Interestingly, in one of the donors, interfering with glycosphingolipid biosynthesis in addition to inhibiting O-glycosylation using Benzyl-α-GalNAc and NB-DGJ, resulted in significant reduction in CT intoxication compared to when only O-glycosylation was inhibited (Fig. 6a and Fig. S7a). However, there was no significant change in binding of either WT CTB or any of the site-specific CTB mutants with enteroids cultured with Benzyl-α-GalNAc versus Benzyl-α-GalNAc + NB-DGJ (Fig. 6b and Fig. S7b, c). The glycosphingolipid analysis showed that there was still no detectable presence of GM1 upon Benzyl-α-GalNAc treatment (Fig. S8), thus the sensitization of enteroids is unlikely due to increase in the high affinity CTB-ligand GM1. There was a slight increase in the other low-affinity binding glycosphingolipids relevant for CTB such as GM2 and GM3, but these were reduced when enteroids were treated with NB-DGJ in addition to Benzyl-α-GalNAc. This suggests that when O-glycosylation is inhibited, even if there was no detectable reduction in CTB binding, the glycosphingolipids might still play a role in CT intoxication because of hetero-multivalent binding (Choi et al. 2019).

Inhibition of fucosylation and O-glycosylation separately sensitized the human enteroids to CT intoxication (Figs 2 and 5), which could also be correlated to the increase in the canonical site binding and increase in the Gal/GalNAc terminated glycoconjugates. However, when fucosylation in addition to O-glycosylation was inhibited, the enhanced intoxication of CT induced by the inhibition O-glycosylation was reversed (Fig. 6a). Interestingly, double inhibition of fucosylation and O-glycosylation didn’t affect the amount of binding to the non-canonical site but rather led to a further increase in the binding of canonical site of CTB (detected by H18L binding) compared to when only O-glycosylation is inhibited (Fig. 6b). This could be partially or completely because of the increased exposure of terminal Gal/GalNAc (detected by PNA binding) which could be binding to the canonical site (Fig. 6c). Therefore, even though the canonical site binding increased potentially because of more Gal/GalNAc terminated glycoconjugates, CT intoxication was rather reduced upon simultaneous inhibition of fucosylation and O-glycosylation. Additionally, inhibition of only O-glycosylation increased surface expression of fucosylated ligands in human enteroids from one of the donors (Fig. 5e). As expected, inhibition of fucosylation drastically diminished any fucose-dependent UEA-1 binding (Fig. 6d).

## Discussion

For several decades, GM1 has been considered as the only binding ligand for CT, that marks the onset of the CT induced diarrheal infection. We have recently shown that not just GM1, but other glycosphingolipids and fucosylated glycoproteins are also potential binding ligands for CT (Wands et al. 2015; Krishnan et al. 2017; Cervin et al. 2018; Lee et al. 2018; Wands et al. 2018; Cervin et al. 2020). However, these results have mainly been demonstrated either in model membranes or human colonic transformed epithelial cells. In this study, we have used a more physiologically relevant model, human enteroids established from jejunal biopsies of several donors, grown as confluent monolayers in the presence of inhibitors of glycoconjugate biosynthesis and estimated CT intoxication by determining the change in the short circuit current from the measurements of transepithelial electrical resistance and potential. We have combined this with assessing the binding of CTB mutants, which have only one of the two binding sites (canonical or non-canonical) functional to investigate the potential role of galactosylated and fucosylated ligands in CT binding to non-transformed human small intestinal epithelial cells.

**Fig. 7:**
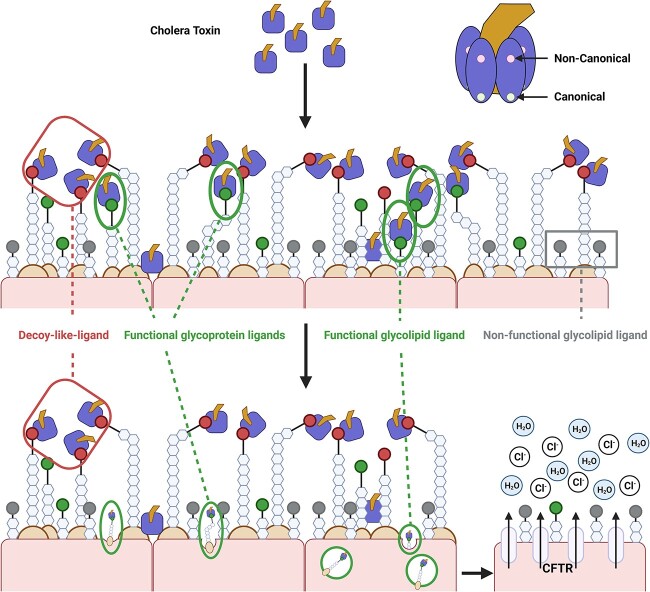
A schematic displaying an intricate interplay between different glycoconjugates (glycosphingolipids and glycoproteins) in CT binding and intoxication. The figure was created with biorender.com.

Collectively, we discovered that inhibition of fucosylation as well as O-glycosylation but not N-linked glycosylation of proteins sensitize non-transformed human enteroids to CT-intoxication. Additionally, glycosphingolipids other than GM1 might be contributing to CT intoxication, which becomes more evident when O-linked glycosylation (potential “decoy-like-ligands”) was inhibited. Our ensuing characterization reveals a potentially intricate interplay between multiple ligands with different functions (schematically summarized in Fig. 7). The sensitization could be a consequence of i) the removal of fucosylated O-linked glycoproteins which act as “decoy-like-ligands” to make other glycoconjugates such as glycosphingolipids more accessible for binding and/or ii) increase in Gal/GalNAc terminated glycoconjugates that bind to the canonical site of CTB and thereby sensitize the treated cells to CT-intoxication. Furthermore, the compensatory increases in Gal/GalNAc glycoconjugate expression caused by inhibition of O-glycosylation and fucosylation are additive in CTB binding but not for intoxication as in the latter, inhibition of fucosylation counteracts the sensitization caused by inhibition O-glycosylation. This implies that besides Gal/GalNAc, fucose could also be an important primary glycan for CT intoxication.

Previous literature have shown that the surface density of GM1 in the human small intestinal epithelium is extremely low (Breimer et al. 2012). We have also previously shown that pre-incubation of CTB with GM1 oligosaccharides did not alter the CTB binding to human enteroids (Cervin et al. 2020). However, L-fucose or fucose binding AAL could efficiently block CTB binding to human jejunal epithelial cells whereas sialic acid binding MAL-II or galactose binding PNA could not (Cervin et al. 2018). We have now shown that GM1 expression is extremely low compared to other glycosphingolipids (Fig. S3). When O-linked glycosylation was inhibited, GM1 was still not detected but the potential role of other glycosphingolipids in CT intoxication was revealed. This further substantiates that other ligands alternate to GM1 are more influential players in CTB binding to and intoxication of non-transformed human small intestinal epithelial cells.

Our CTB mutants binding data reveal that most of the ligands on non-transformed human small intestinal epithelial cells bind to the non-canonical site and only a few ligands bind to the canonical site. This can be corroborated by a previous observation that epithelial cells highly express Lewis-x and that CTB binding to primary jejunal epithelial cells could be efficiently blocked by Lewis-x binding to the non-canonical site (Cervin et al. 2018; Heim et al. 2019). Interestingly, the sum of the individual binding to both the canonical and non-canonical sites do not add up to the overall cumulative binding of WT CTB. This discrepancy is not because of the incapability of the mutants to perform with an unimpaired binding site as H18L binding to GM1 is comparable to WT CTB (Fig. 3a) and W88K binding to Lewis-x is comparable to WT CTB (Fig. 3b). Two potential reasons that could explain this discrepancy are: a) allosteric interactions between the two sites (Changeux 2013), and/or b) heteromultivalent cooperative interactions between ligands via the reduction in dimensionality (Krishnan et al. 2017; Choi et al. 2019; González-Cuesta et al. 2020). However, to distinguish which of these interactions play a more dominant role in governing the CTB binding is beyond the scope of this research. Binding of toxin to the ligands on cell membrane may not always result in toxin internalization, or internalization that is on-pathway to intoxication. It is well established that cell surface biology is mediated by multivalent interactions between low-affinity proteins and glycans (Collins and Paulson 2004). In addition to valency, membrane fluidity influences the overall binding avidity (Krishnan et al. 2017; Choi et al. 2019). Higher surface density is needed for low-to-moderate affinity binding ligands to facilitate toxin internalization whereas high-affinity ligands could achieve the same with a lower surface density and this could be further influenced by the relative surface density of high- and low-affinity ligands (Alves et al. 2011; Fakhari et al. 2011; Hadzhieva et al. 2017; Csizmar et al. 2019). Thus, this interplay between the ligand surface density and binding affinity categorizes the role of each glycoconjugate as functional or decoy like activity against pathogens. Additionally, targeting just one site on a toxin may not be sufficient for antidote or vaccine development. Our data reveals that fucosylated ligands binding to the non-canonical site may act as “decoy-like ligands” whereas fucosylated and galactosylated ligands binding to the canonical site may act as functional ligands. This is also supported by our previous observation that a mixed polymer with fucose and galactose could block CTB binding and CT intoxication to human enteroids more efficiently than only fucosylated or galactosylated polymers (Cervin et al. 2020).

Furthermore, a recent perspective that has been proposed is that most glycan functions are “analog” and not “digital” i.e. most intrinsic glycan functions are mediated by a spectrum of structures in contrast to amino acids or nucleic acids (Varki 2017). Host cells undergo subtle alterations in glycosylation without drastically affecting the intrinsic functions, to escape pathogens and remain relevant in the process of evolution. Thus, a more nuanced approach needs to be used when analyzing alterations in glycosylation. For example, we unraveled two of the possible outcomes with inhibitors, Benzyl-α-GalNAc and 2F-Fuc—a) elimination of “decoy-like-ligands” making it easier for CTB to access and bind to other functional ligands closer to the cell surface, b) increased availability of glycoconjugates binding to the canonical site and contributing to CT intoxication. Increase in the surface availability of Gal/GalNAc ligands upon inhibition of fucosylation in human enteroids was an unexpected outcome. This makes it difficult to conclude that if the sensitization of enteroids to CT intoxication was a result of downregulation of decoys or upregulation of some functional ligands or both. This is also the limitation of using inhibitors to modulate glycosylation as although they cause reduced expression of specific glycans, compensatory increased expression of other classes of glycans could occur. A potential alternative is to enzymatically cleave glycan structures on a fixed cell surface. This prevents compensatory alterations in glycoconjugate expression but has the obvious limitation of not allowing assays that require viable cells. In addition, as membrane fluidity plays an essential role in facilitating binding interactions (Krishnan et al. 2017), the loss of fluidity of glycoconjugates as a result of cell fixation might underestimate the overall binding occurring at the membrane surface. Thus, it is challenging to choose an ideal method. Nonetheless, the use of these inhibitors could be a useful tool to study alterations in glycosylation and the results from our functional assays will need to be complemented with additional biochemical approaches. Furthermore, this phenomenon of upregulation of Gal/GalNAc ligands with Benzyl-α-GalNAc and 2F-Fuc is not unique to human enteroids as similar results can be seen in colonic epithelial cell line such as Colo cells (Fig. S9).

One potential group of “decoy-like ligands” on the cell surface that could limit the pathogenesis of CT are transmembrane mucins. These are heavily O-glycosylated (core 3 structure mucins in the small intestine) that are known to mimic the potential glycan ligands in the glycocalyx to bind to pathogens as a part of the immune system to limit the pathogen binding to other glycoconjugates (Robbe et al. 2004; McGuckin et al. 2011; Dennis and Brewer 2013; Ringot-Destrez et al. 2018; Amimo et al. 2021). In addition to O-glycosylation, mucins comprise of a much smaller fraction of N-linked glycans which are mainly implicated in surface localization (Linden et al. 2008). This correlates well with our data that inhibition of N-linked glycosylation has little effect on CT mediated intoxication of enteroids. The similar phenomenon of sensitization of human enteroids to CT intoxication in the absence of O-glycosylation was observed during inhibition of fucosylation. Since mucin chains contain fucose (Linden et al. 2008), it is plausible that O-glycosylated mucins bind to CTB via fucose glycans and thereby act as decoys.

Finally, it is unlikely that the only role of fucose in CT intoxication is facilitating the decoy like function. Inhibition of fucosylation in addition to O-glycosylation rather than further sensitizing, abrogated the effect of enteroid sensitization towards CT intoxication caused by Benzyl-α-GalNAc (Fig. 6a). This suggests that in addition to terminal Gal/GalNAc, fucose may also functionally contribute to CT intoxication. A possible explanation could be that fucose on O-linked glycans may act as a decoy glycan while fucose on other glycoconjugates such as glycosphingolipids may act as functional glycan in context of CT intoxication. Crystallographic studies have shown that CTB binds to most fucosylated structures via the non-canonical site except for fucosyl-GM1 glycosphingolipid where the canonical site is employed (Heim et al. 2019). We hypothesize that it depends on whether fucose is present on O-linked glycoproteins, it might be contributing to decoy like behavior or if it is present on glycosphingolipids, it might have a more functional role in CT intoxication. Such functional fucosylated ligands could be histo-blood group antigens expressed on the intestinal epithelial cells (Holmner et al. 2004; Heggelund et al. 2016). This could also provide explanation to the selective severity of cholera infections in people with O blood group with terminal fucosylated H-antigen (Swerdlow et al. 1994; Mandal et al. 2012; Kuhlmann et al. 2016) which has possibly resulted in natural selection against cholera as the lowest prevalence of blood group O individuals are found in the Ganges River Delta where cholera is still an endemic (Glass et al. 1985; Karlsson et al. 2013; Kumar et al. 2018). Hence, this study has unraveled potential dual functions of glycoconjugate ligands which are not GM1 and are expressed on non-transformed human small intestinal epithelial cells. These findings could further the understanding and help to decipher the differences in diarrheal response to *Vibrio cholerae* infection and thereby facilitate the design of novel antidotes.

## Materials and methods

### Establishment of human enteroids

Organoids were prepared as described before (Cervin et al. 2020). Briefly, small biopsies of the human jejunum were treated with antibiotic-antimyotic (Thermo Scientific) in PBS (1:100) for 2 mins, repeated 4× and then with 10 mM DTT (Fisher Scientific) in PBS for 2 mins, repeated 3×. The tissue was then incubated with 2 mM EDTA (Sigma Aldrich) in PBS at 4 °C for 1 h and then shaken vigorously in rounds of PBS to isolate the crypts. The crypts were then seeded into Matrigel, hESC-Qualified Matrix (Corning) and cultured in human Intesticult Organoid Growth Medium (STEMCELL). The blood group of each donor was unknown.

### CT intoxication of human monolayers

Established enteroids were dissociated in TrypLE express (Gibco) at 37 °C for 45 min. The resulting single cell suspensions were seeded into transwell membranes (Corning Transwells – clear polyester membranes 0.4 μm pore 0.33 cm^2^) coated with collagen from human placenta (Sigma-Aldrich) and grown in a 1:1 mixture of Human Intesticult Organoid Growth Medium (OGM, STEMCELL) and Human Intesticult Differentiation Medium (DM, STEMCELL) and replaced with fresh media every 2 days until confluency. Monolayer confluency was assessed every 2–3 days via microscopy and transepithelial electrical resistance (TEER) measurements readings using Millicell ERS-2 Volt-Ohm meter (Millipore). When the monolayers had reached confluency and the TEER had reached 600 ohms/cm^2^, the cells were grown in DM for 2 days after which the media was replaced by in DM containing inhibitors for another 3 days. The final concentration of inhibitors NB-DGJ (50 μM, N-(n-Butyl)deoxygalactonojirimycin, Sigma Aldrich), 2F-Fuc (200 μM, 2F-Peracetyl-Fucose, Sigma Aldrich), Benzyl-α-GalNAc (2 mM, Benzyl-2-acetamido-2-deoxy-α-d-galactopyranoside, Sigma Aldrich), P4 (1 µM, DL-threo-PDMP, Hydrochloride, Sigma Aldrich) and Kifunensine (10 μg mL^−1^, Sigma Aldrich) was decided based on if there was no significant cell death with inhibitors compared to when no inhibitors were present. After 3 days, the monolayers were challenged with 2 μg mL^−1^ CT (List Labs) on the apical side. The TEER and voltage were recorded each hour to calculate the short circuit current (${I}_{sc, CT}^t$) to evaluate ion efflux. To compare the inter-day results, the measurements at each time point were then normalized as:


$$ {I}_{sc, CT}^1 = {I}_{sc, CT}^{t} \Big/ {I}_{sc, CT}^{t=0} $$

$$ {I}_{sc}={I}_{sc, CT}^t-{I}_{sc, no\ CT}^t $$

### Synthesis of CTB mutants

All bacterial strains used in this study were maintained on modified syncase agar plates. Bacterial DNA alterations were performed according to the Thermo Fisher Scientific protocols. Primers and the sequencing service for bacterial plasmids were provided by Eurofins Genomics.

The W88K mutant was constructed via PCR primer extension using the pML-ctxB thyA plasmid from strain JS1569 (*Vibrio Cholerae* cells). For this, custom forward and reverse CTB-W88K specific primers were used in combination with a reverse or forward BspEI primer respectively, producing two halves of the entire plasmid with the W88K mutation. The fragments were then cleaned using the GeneJET PCR Purification Kit (Thermo Scientific) and run in a primerless PCR reaction using Dreamtaq polymerase (Thermo Scientific). Subsequently, BspEI forward and reverse primers were added to the reaction mixture with fresh Dreamtaq polymerase for another PCR reaction. The PCR product was digested with a BspEI restriction enzyme in Tango buffer. The resulting DNA was cleaned and ligated with T4 ligase (Thermo Scientific) and electroporated into electrocompetent cells from the strain JS1569 4.4 and recovered in modified syncase media prior to plating. Successful plasmid transfer was confirmed via sequencing (Eurofins Genomics).

A custom DNA reverse primer and sense strand were designed to create random single base pair mutations at H18 in the CTB gene. The synthesized DNA strand was first annealed with the reverse primer at a 1:1 ratio in 1x T4 Polymerase buffer (NEB) overnight at 4 °C. The following day the mixture had dNTPs and T4 Polymerase (NEB) added and incubated at 4 °C for 20 min, stopping the reaction by incubating at 75 °C for 20 min. The DNA was then purified using the GeneJET PCR purification Kit and run on a gel to confirm. The pML-ctxB/thyA plasmid was digested with SacI and Bstz171 restriction enzymes (FastDigest—Thermo scientific) at 37 °C for 15 min. The synthesized H18 mutated strands were also restriction digested in the same way. The plasmid DNA was then dephosphorylated using FastAP (Thermo Scientific) according to the protocol and cleaned as before alongside the cut synthesized DNA fragment. The synthesized fragment was then ligated into the plasmid using T4 DNA ligase and electroporated into electrocompetent JS1569 4.4 cells and recovered in modified syncase media prior to plating. Colonies were picked and restreaked and further had their plasmids isolated for DNA sequencing.

For the H18LW88K mutant, the plasmids from the H18L and W88K mutants were isolated and cut with SacI and Bstz171. The W88K plasmid was then dephosphorylated and both plasmid restrictions were cleaned. As previously described, the H18L fragment was then ligated into the W88K plasmid and electroporated into electrocompetent JS1569 4.4 cells. Successful incorporation was identified by DNA sequencing.

### Mutant CTB protein purification and biotinylation

CTB mutant expressing bacterial strains were inoculated from a pre-culture and cultured overnight in modified syncase media in a large volume at 37 °C. Bacterial broths were then centrifuged at 10,150 × g for 30 min to pellet bacteria where the supernatant was saved. Sodium hexametaphosphate was added at a concentration of 2.5 g L^−1^ to the CTB media, and the pH adjusted to 4.5 for WT and W88K, or 3.5 for H18L and H18LW88K mutations, where the mixtures were left to stir overnight at 4 °C. Supernatants were spun again at 10,150 × g for 60 min at 4 °C. The resulting precipitate was resuspended in Tris-HCL pH 7.5, where it went through 2 rounds of dialysis in 6,000–8,000 MWCO tubing (Spectrum) for 8 h. The resulting protein sample was run through FPLC via an affinity chromatography column (Resource-Q 6 mL) using Tris-HCL pH 8.0 start buffer, with Tris-HCL + 1 M NaCl as an elution buffer.

The CTB mutants’ concentration were determined by nanodrop. The mutants at 1.8 mg mL^−1^ were then biotinylated using Type A Biotinylation kit Lightning-Link (Abcam).

### ELISA

100 μL of GM1-HSA (0.1 μg mL^−1^, IsoSep) or tri-LeX-APE-HSA (10 μg mL^−1^, IsoSep) in PBS were added to high binding microplates (Greiner) and left to incubate overnight at 4 °C for immobilization. Plates were then washed with 0.2% (w/v) BSA (Sigma-Aldrich) in PBS at 37 °C. The plates were then incubated with titrations of each CTB mutant in PBS + 0.2% BSA + 0.05% Tween20 at room temperature for 1 h, followed by rabbit anti-CTB primary antibody (Sigma Aldrich) incubation for 90 mins at room temperature. Anti-rabbit-IgG-HRP secondary antibody (Jackson ImmunoResearch) was then added and incubated for 2 h. Finally, 1-Step Ultra TMB-ELISA substrate solution (Thermo Scientific) was added and followed by addition of sulfuric acid (Sigma Aldrich) to detect the overall binding. The plates were read using ELx800 (BioTek) at 450 nm. The binding of non-biotinylated and biotinylated CTB mutants to GM1-HSA and tri-LeX-APE-HSA were comparable.

### Cell culture

Jurkat cells and Colo205 cells were obtained from the ATCC. The cells were grown in the incubator at 37 °C and 5% CO_2_, in media according to the ATCC recommended media compositions. The adherent Colo205 cells were suspended in solution by incubating with TrypLE express (Gibco) for 40 mins followed by inactivation with DMEM +10% heat-inactivated Fetal Bovine Serum (FBS, Gibco). The resuspended Colo205 and Jurkat cells were then counted and reseeded at the recommended density.

### Mice

Mice from the C57BL/6 background which are +/− (WT) and −/− (KO) for the B4galnt1 gene were kindly donated by Professor Ronald L. Schnaar (John Hopkins University School of Medicine, Baltimore, MD, USA). The −/− (KO) mice are unable to generate complex and most noncomplex glycosphingolipids (Sheikh et al. 1999). The mice were bred using a heterozygous-homozygous breeding strategy and kept in ventilated cages at the animal facility on site at Sahlgrenska Academy, University of Gothenburg, Sweden. All experiments were performed in accordance with the approved ethical permit granted by the regional animal’s ethics committee (ethics no. 1092/17).

Splenocytes were isolated from the spleens of sacrificed WT and KO mice by gentle mashing through a 100 μM filter. The resulting cell mixture was then lysed using Red Blood cell lysing buffer (BD) to remove the majority of the red blood cells and the cells were used for Flow Cytometry staining.

### Flow cytometry: CTB mutants and lectin binding

Established enteroids were suspended in 1:1 mixture of OGM and DM for 2 days and then DM containing inhibitors (same concentration as CT intoxication experiment) for 3 days to obtain everted enteroids with (apical side out). The everted enteroids were then made into single cell suspensions using TrypLE at 37 °C for 40 mins before staining.

Colo205 cells were similarly treated with TrypLE express at 37 °C for 40 mins prior to staining. Granulocytes were generously donated from buffy coat Ficoll density gradient separations. Cells were then stained for flow cytometry to determine gMFI binding of each CTB mutant.

The staining with human enteroids, murine splenocytes, human granulocytes and cell lines, Jurkat and Colo205 were performed at 4 °C in the presence of FACS buffer (PBS + 1 mM EDTA +2% FBS). Isolated single cells were stained with the live/dead marker Aqua (Invitrogen, Fisher Scientific) for 10 mins, followed by washing with FACS buffer 2×. The enteroids were then incubated with either 1.25 μg mL^−1^ biotinylated CTB mutants or lectins, 10 μg mL^−1^  *Ulex Europaeus* Agglutinin I biotinylated (UEA-1, vector labs), 20 μg mL^−1^ Peanut Agglutinin biotinylated (PNA, vector labs), 20 μg mL^−1^  *Maackia Amurensis* Lectin II (MAL II, vector labs) for 30 mins or 20 μg mL^−1^  *Sambucus Nigra* Lectin (SNA, vector labs) for 20 mins, followed by washing with FACS buffer 2x before incubation with 1 μg mL^−1^ Streptavidin-APC (BioLegend). The cells were then washed with FACS buffer 2x and then data was acquired using a flow cytometer (LSRII, BD) and analyzed using FlowJo 10 (FlowJo LLC, BD).

In the experiments involving *Vibrio cholerae* neuraminidase (VcN, Sigma Aldrich) treatment, Jurkat cells or human enteroids were incubated with 100 mU mL^−1^ at 37 °C for 60 mins, after which the single cells were isolated and stained as mentioned above.

### HPLC

The method for GSL analysis is described in a previous publication (Huebecker et al. 2019). The enteroid pellets were washed with PBS, 0.5 mL water added and freeze-thawed 3×, before following the published GSL protocol (https://www.protocols.io/view/analysis-of-glycosphingolipids-from-human-plasma-busvnwe6).

## Supplementary Material

Supplementary_Information_cwad069

## Data Availability

The data underlying this article will be shared on reasonable request to the corresponding authors.
