# Supplementary material for: Cholera intoxication of human enteroids reveals interplay between decoy and functional glycoconjugate ligands
Source: Glycobiology. 2023 Aug 25;33(10):801–16. doi: 10.1093/glycob/cwad069 (PMC10629719; doi:10.1093/glycob/cwad069)
Supplement: Supplementary_Information_cwad069 [file supplementary_information_cwad069.docx]

# Supplementary Information

# Cholera intoxication of human enteroids reveals interplay between decoy and functional glycoconjugate ligands

Akshi Singla^1,2^*, Andrew Boucher^1^, Kerri-Lee Wallom^3^, Michael Lebens^1^, Jennifer J Kohler^4^, Frances Platt^3^, and Ulf Yrlid^1^*

^1^ Department of Microbiology and Immunology, Institute of Biomedicine, University of Gothenburg, 405 30 Gothenburg, Sweden

^2^ Department of Medical Chemistry and Cell Biology, Institute of Biomedicine, University of Gothenburg, 405 30 Gothenburg, Sweden

^3^ Department of Pharmacology, University of Oxford, Oxford, UK

^4^ Department of Biochemistry, University of Texas Southwestern Medical Center, Dallas, Texas, USA

* Corresponding authors

Akshi Singla: [akshi.singla@gu.se](mailto:akshi.singla@gu.se)

Ulf Yrlid: [ulf.yrlid@microbio.gu.se](mailto:ulf.yrlid@microbio.gu.se)

**Short running head**: Interplay between decoy & functional ligands: Cholera toxin

**Keywords**: Cholera toxin / Decoy-like-ligands / Enteroid monolayers / Fucosylation / O-Glycosylation


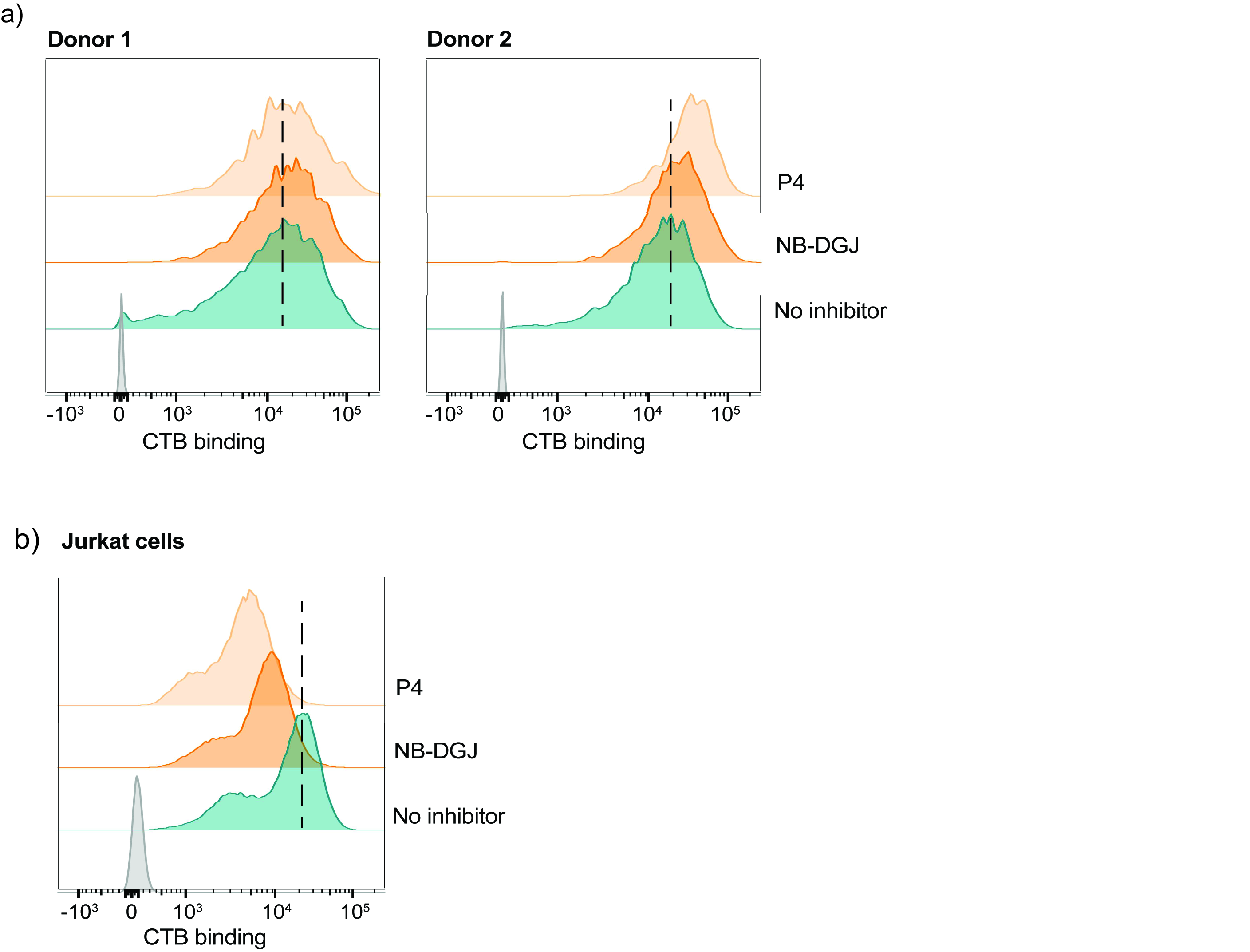


*SI Figure 1: Cell surface binding of CTB to a) human enteroids and b) Jurkat cells measured by flow cytometry.*

*
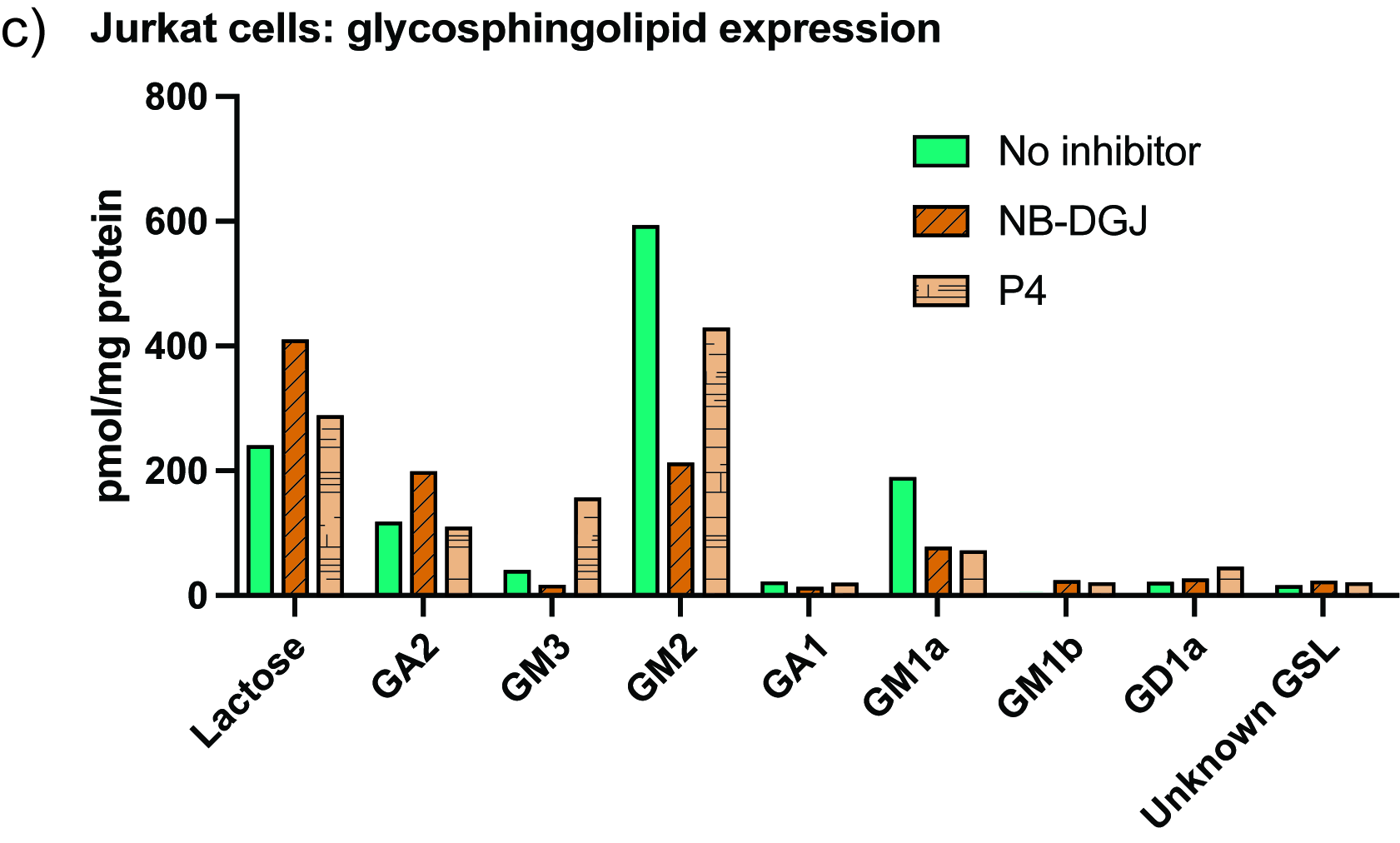
*

*SI Figure 2: Individual glycosphingolipid expression measured by HPLC in GM1 rich Jurkat cells before and after NB-DGJ & P4 treatment.*


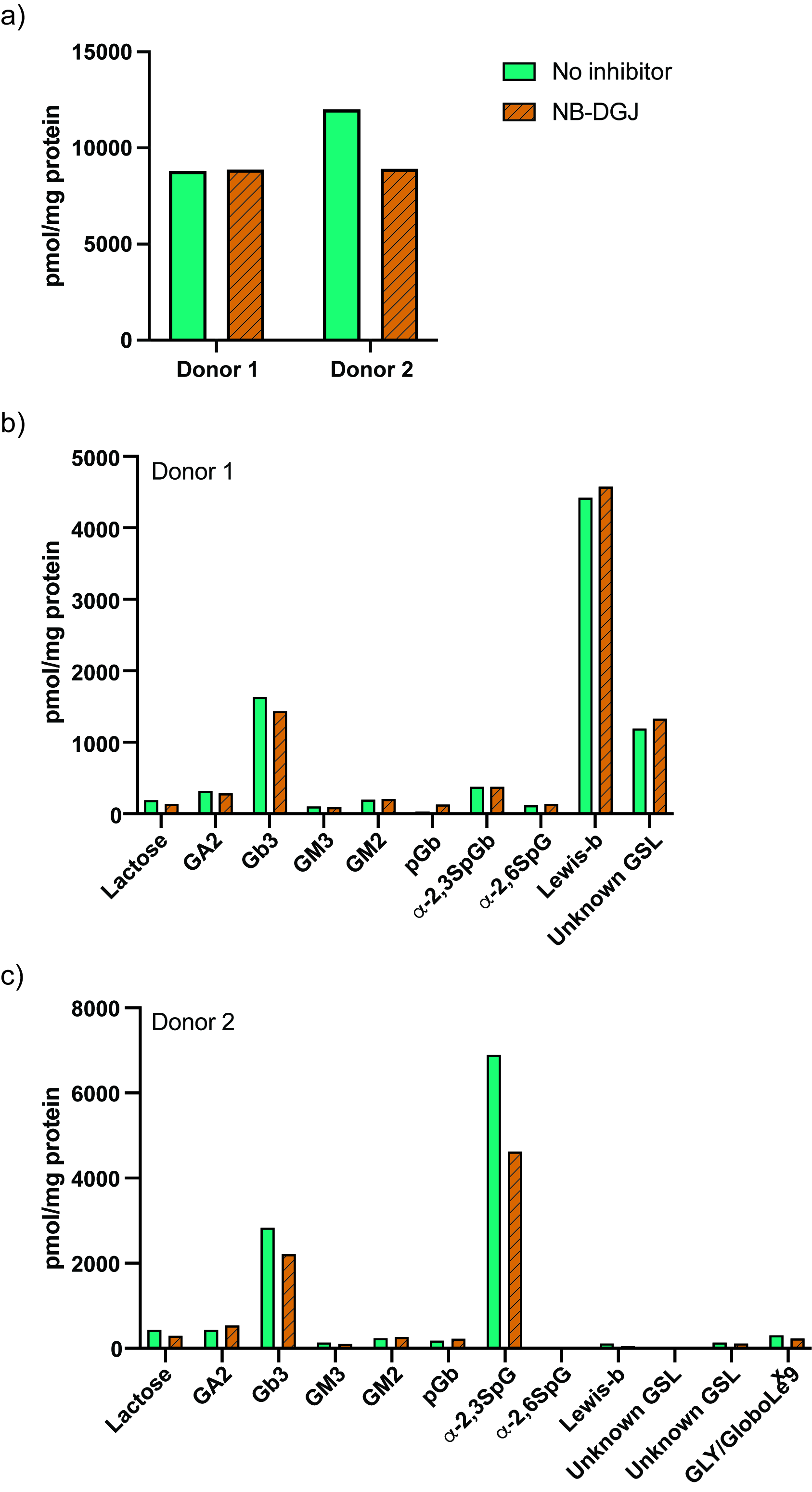


*SI Figure 3: Glycosphingolipid expression measured by HPLC in human enteroids before and after NB-DGJ treatment. a) total expression, b&c) Expression of individual glycosphingolipid.*


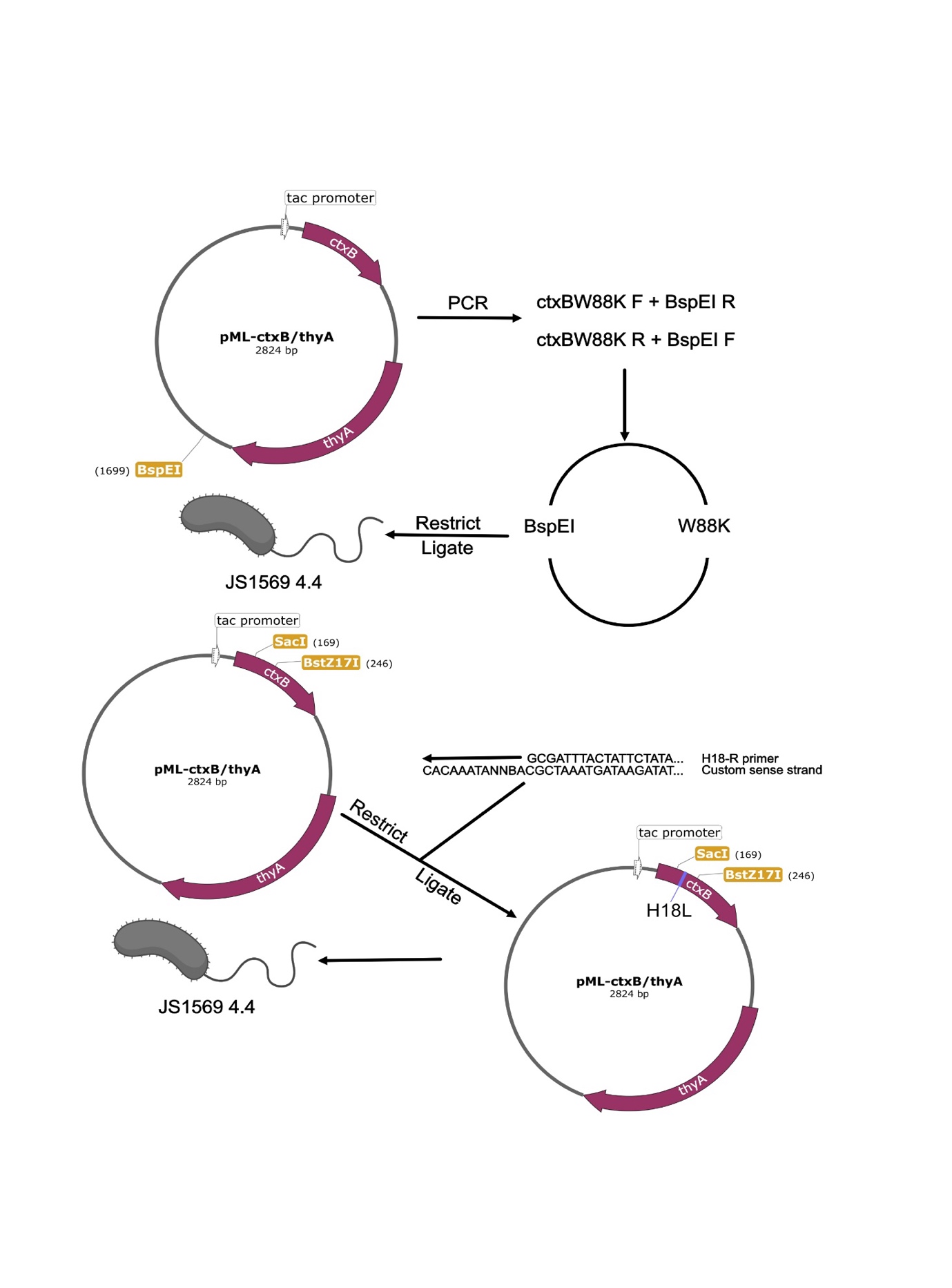


**A**

**B**

*SI Figure 4: Scheme showing the stages for the construction of the (A) W88K and (B) H18L mutants of CTB. (A) The W88K mutation was introduced using the plasmid pML-ctxB/thyA by amplification using custom primers at the W88 and BspEI sites to create two plasmid halves containing the mutation. The two halves were joined by primerless PCR and subsequently amplification with the two BspEI primers. The products were ligated to form a full circular plasmid and transformed into the electrocompetent strain JS1569 4.4. (B) A custom sense strand designed to have random nucleotide mutations at position H18 was PCR amplified with a reverse primer. The plasmid pML-ctxB/thyA and the newly synthesized H18L containing double strand were cut with SacI and BstZ17I. The products were ligated and transformed into JS1569 4.4*


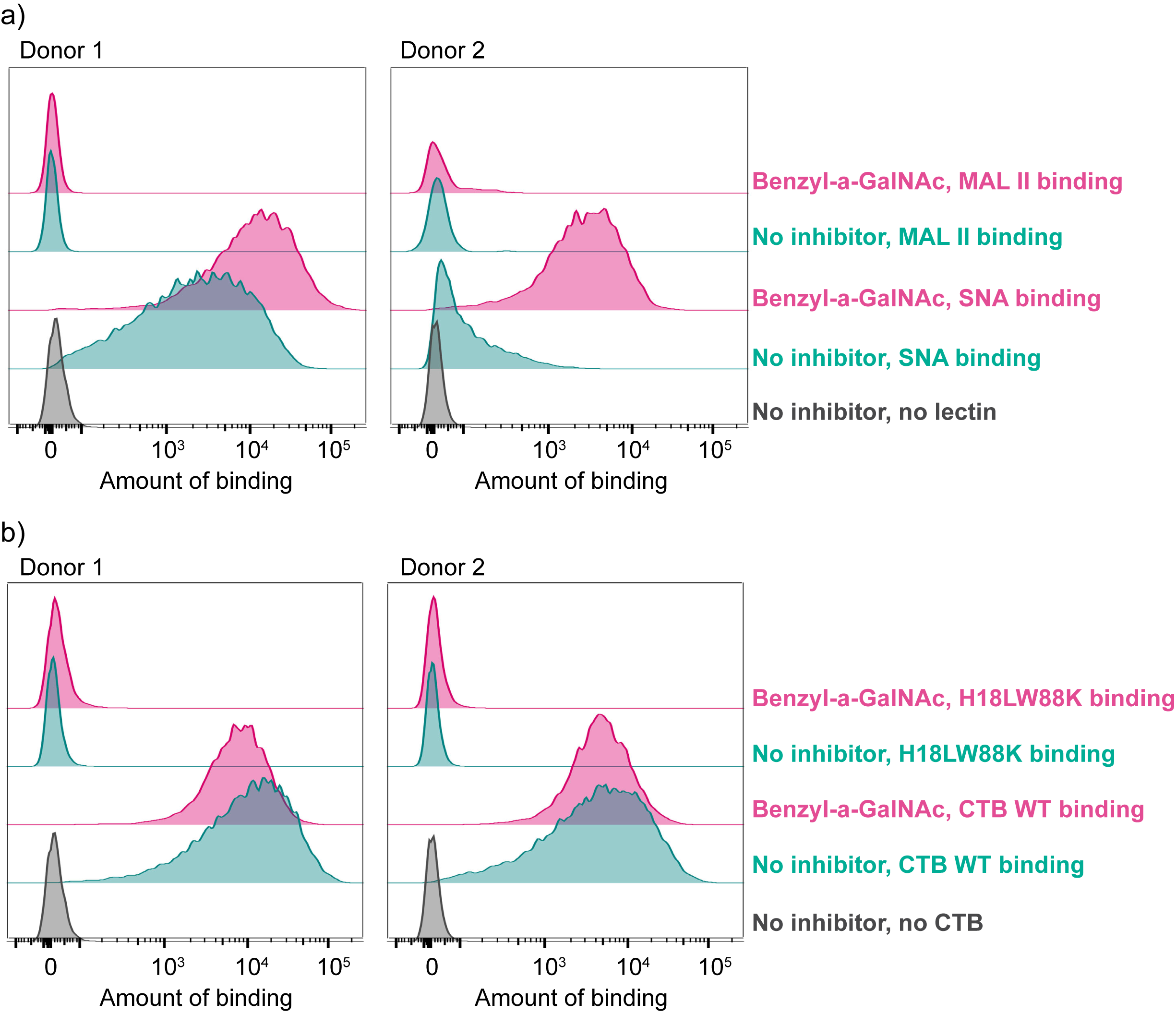


*SI Figure 5: Role of sialylation on CTB binding to human enteroids upon Benzyl-α-GalNAc treatment as assessed by flow cytometry. a) sialic acid specific lectin binding to human enteroids (MAL II binds to α-2,3 linked sialic acid and SNA binds to α-2,6 linked sialic acid), b) CTB WT and H18LW88K mutant binding to human enteroids.*

*
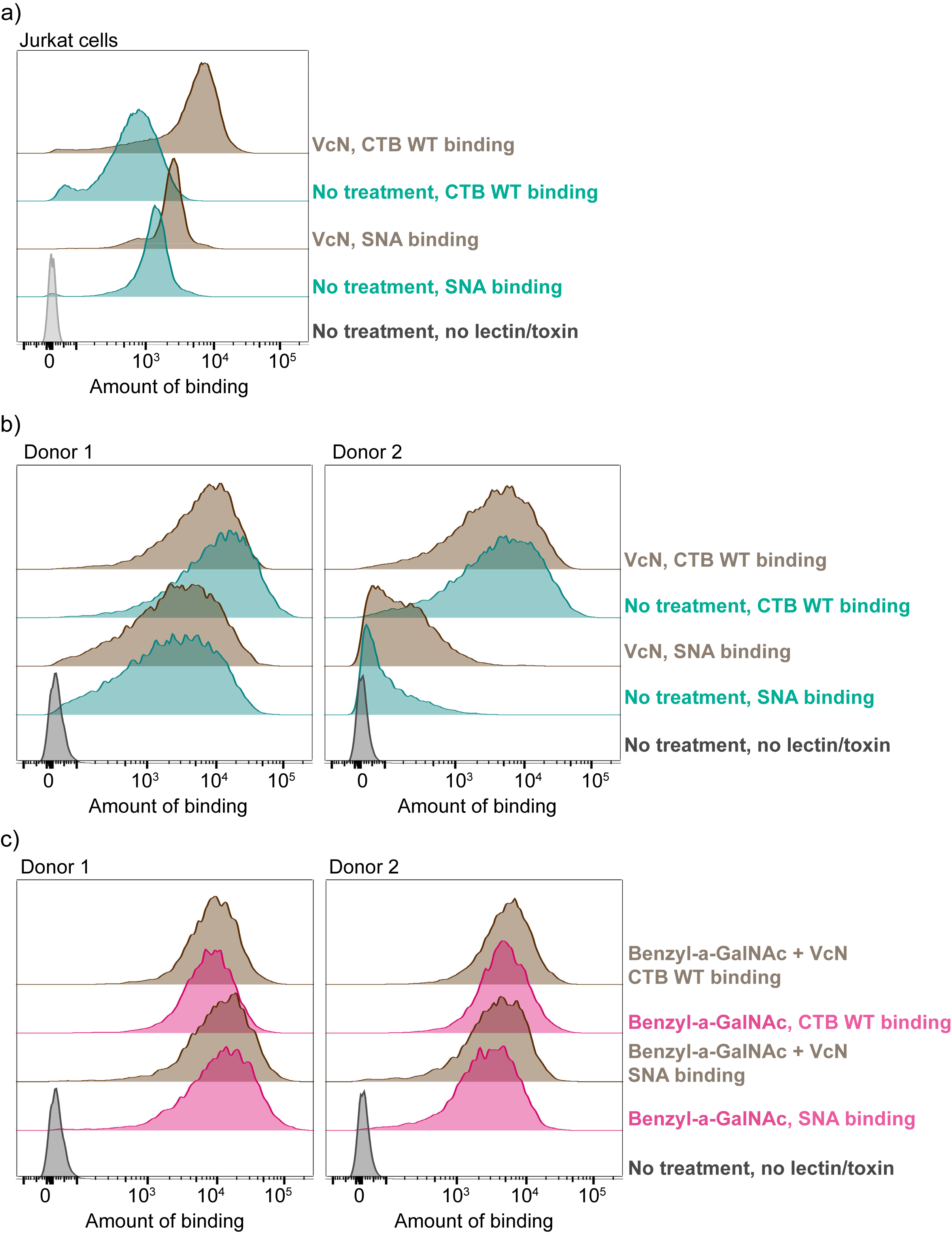
*

*SI Figure 6: Effect of VcN on sialylation and CTB mutant binding as assessed by flow cytometry, a) Jurkat cells b) human enteroids, c) human enteroids treated with Benzyl-α-GalNAc treatment.*


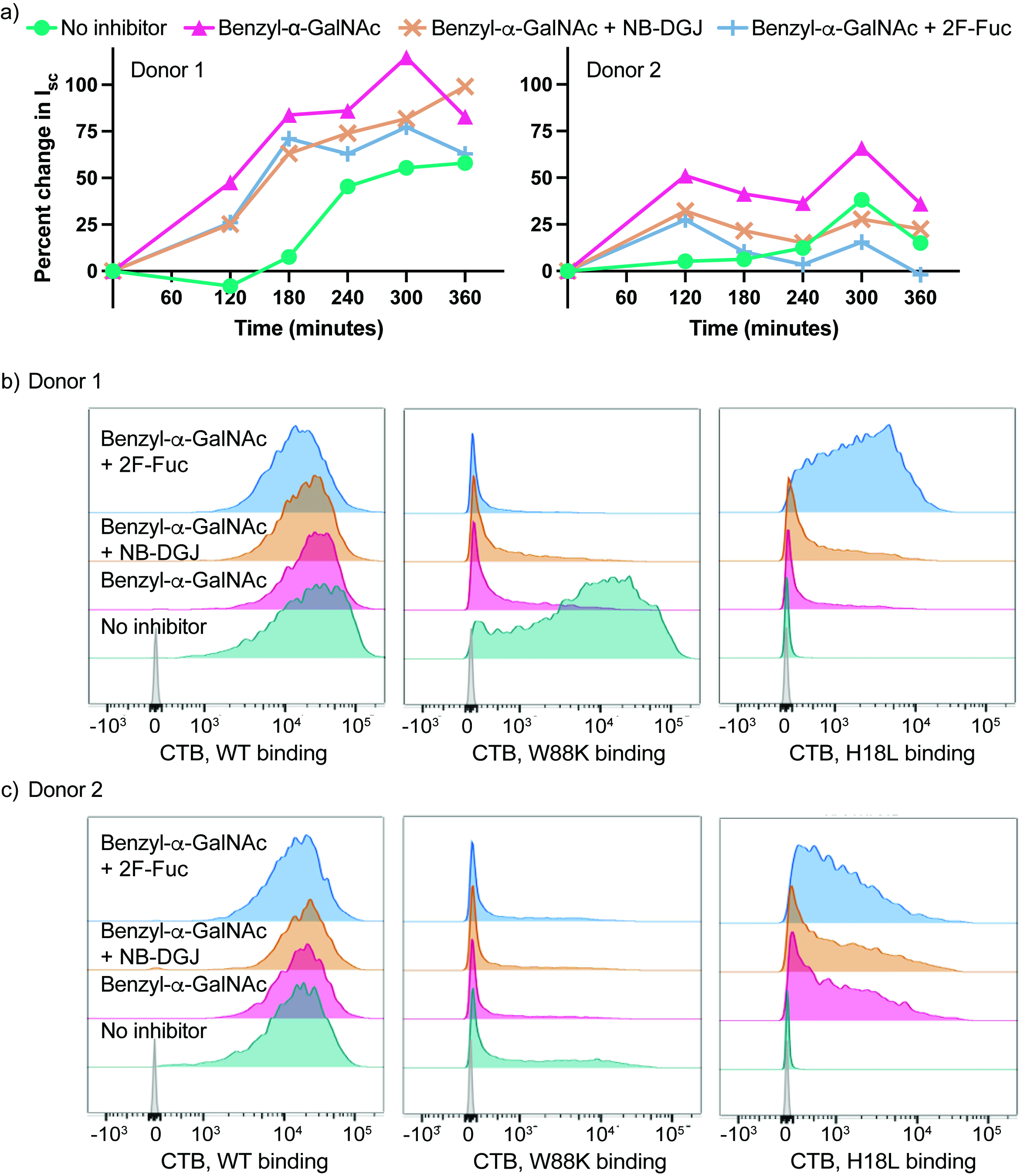


SI Figure 7: Role of glycolipids and fucosylation in the absence of “decoy-like-ligands”. a) Rate of CT-mediated intoxication of enteroid monolayers. The data shown is a representative of n = 3 independent experiments. b&c) Binding of CTB mutants to human enteroids measured via flow cytometry. The data shown is a representative of n = 3 independent experiments.


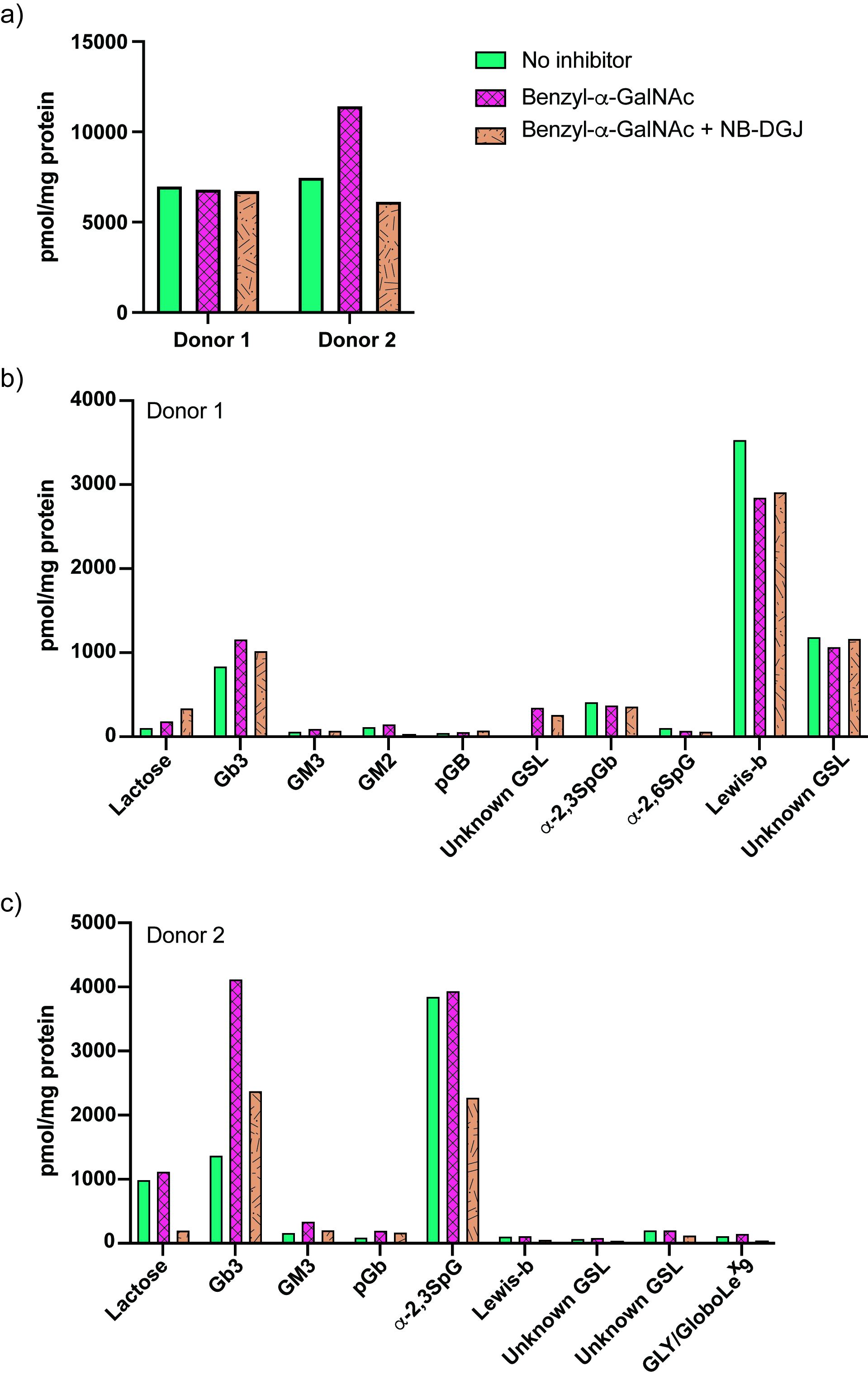


*SI Figure 8: Glycosphingolipid expression measured by HPLC in human enteroids before and after Benzyl-α-GalNAc and Benzyl-α-GalNAc + NB-DGJ treatment. a) total expression, b&c) Expression of individual glycosphingolipid*


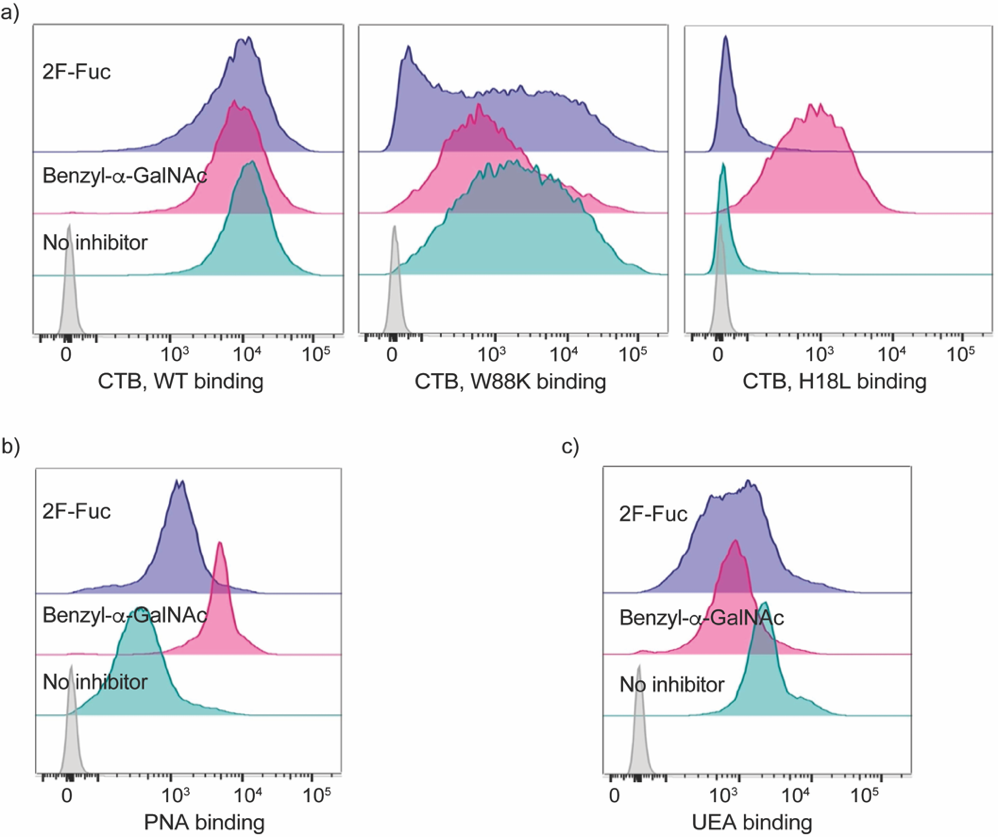


SI Figure 9: Colo 205 cells display a similar binding pattern as enteroids to CTB-mutants and lectins.
